# Effect of Climate Change on CO_2_ Flux in Temperate Grassland, Subtropical Artificial Coniferous Forest and Tropical Rain Forest Ecosystems

**DOI:** 10.3390/ijerph182413056

**Published:** 2021-12-10

**Authors:** Zihao Man, Shengquan Che, Changkun Xie, Ruiyuan Jiang, Anze Liang, Hao Wu

**Affiliations:** School of Design, Shanghai Jiao Tong University, Shanghai 200240, China; manzihao1023@163.com (Z.M.); xiechangkun@sjtu.edu.cn (C.X.); jiangry@sjtu.edu.cn (R.J.); 018150210001@sjtu.edu.cn (A.L.); wuhao1101@sjtu.edu.cn (H.W.)

**Keywords:** CO_2_ flux, gross ecosystem exchange, ecosystem respiration, temperature, moisture, climate change, carbon neutrality

## Abstract

The interactions between CO_2_ flux, an important component of ecosystem carbon flux, and climate change vary significantly among different ecosystems. In this research, the inter-annual variation characteristics of ecosystem respiration (RE), gross ecosystem exchange (GEE), and net ecosystem exchange (NEE) were explored in the temperate grassland (TG) of Xilinhot (2004–2010), the subtropical artificial coniferous forest (SACF) of Qianyanzhou (2003–2010), and the tropical rain forest (TRF) of Xishuangbanna (2003–2010). The main factors of climate change affecting ecosystem CO_2_ flux were identified by redundancy analysis, and exponential models and temperature indicators were constructed to consider the relationship between climate change and CO_2_ flux. Every year from 2003 to 2010, RE and GEE first increased and then decreased, and NEE showed no significant change pattern. TG was a carbon source, whereas SACF and TRF were carbon sinks. The influence of air temperature on RE and GEE was greater than that of soil temperature, but the influence of soil moisture on RE and GEE was greater than that of air moisture. Compared with moisture and photosynthetically active radiation, temperature had the greatest impact on CO_2_ flux and the exponential model had the best fitting effect. In TG and SACF, the average temperature was the most influential factor, and in TRF, the accumulated temperature was the most influential factor. These results provide theoretical support for mitigating and managing climate change and provide references for achieving carbon neutrality.

## 1. Introduction

Ecosystem CO_2_ flux refers to the net amount of CO_2_ absorbed and released by the carbon cycle in the ecosystem. It depends on the net ecosystem exchange (NEE), which is the difference between the gross ecosystem exchange (GEE) and the ecosystem respiration (RE) [1,2,3]. The ecosystem sequesters CO_2_ through photosynthesis and other methods, and respiration releases CO_2_ back into the atmosphere [4,5,6,7]. If GEE is greater than RE, the ecosystem acts as a carbon sink; otherwise, it is a carbon source [8,9,10]. There are many factors that affect CO_2_ flux, such as temperature, precipitation, radiation, and soil properties [11,12,13]. However, the effects of these factors vary among different ecosystems [14,15,16,17]. In arid areas, precipitation can increase soil moisture and promote photosynthesis and ecosystem respiration, while warming can reduce soil moisture and the rate of photosynthesis and respiration [18,19]. In humid areas, precipitation causes a drop in soil temperature and reduces oxygen availability in the soil, leading to the weakening of photosynthesis and respiration, while warming increases soil temperature and promotes the rate of photosynthesis and respiration [20,21]. In addition, the increase in precipitation and temperature has been shown to lead to the change in soil temperature and moisture, affecting the soil vegetation and microbial activities and altering the ecosystem CO_2_ flux [21,22].

With the impact of human activities and urban expansion, the concentration of CO_2_ and other greenhouse gases has increased several times relative to before the industrial revolution. Correspondingly, CO_2_ emissions have become one of the strongest factors influencing global climate change [23,24,25,26]. The process of climate change has accelerated because of the continuous emission of CO_2_ and other greenhouse gases. The circumstances, such as rising temperature, increased the intensity of rainstorms and drought, and increased frequency of rapid turn from drought to flood, were threatening ecosystems and even social development [27,28,29,30]. However, various ecosystems have different abilities to resist climate change, and the impact of meteorological factors on different ecosystems is also variable. These differences lead to considerable variations in CO_2_ fluxes among different ecosystems under the influence of climate change. Therefore, it is important to clarify the differences in CO_2_ fluxes among various ecosystems and understand the relationship between climate change and the ecosystems for the protection of ecosystem biodiversity and the mitigation and governance of global climate change.

At present, the mechanism of ecosystem CO_2_ flux on climate change remains uncertain, particularly how climate change will influence ecosystem CO_2_ flux [31] and what changes will occur in carbon cycling. Moreover, the question of how to best achieve the goal of carbon neutrality remains unanswered; however, this has become a major topic of international research and technological development. In this study, RE, GEE, NEE, soil and air temperature, soil and air moisture, and photosynthetically active radiation data were examined for temperate grassland (TG) in Xilinhot, subtropical artificial coniferous forest (SACF) in Qianyanzhou, and tropical rain forest (TRF) ecosystems in Xishuangbanna from 2003 to 2010. There were three main objectives: (1) to analyze the inter-annual variation characteristics of RE, GEE, and NEE for the three ecosystems; (2) to identify main factor of climate change affecting ecosystem CO_2_ flux; and (3) to reveal the mechanism of climate change on ecosystem CO_2_ flux.

## 2. Materials and Methods

### 2.1. Study Area

**Figure 1:**
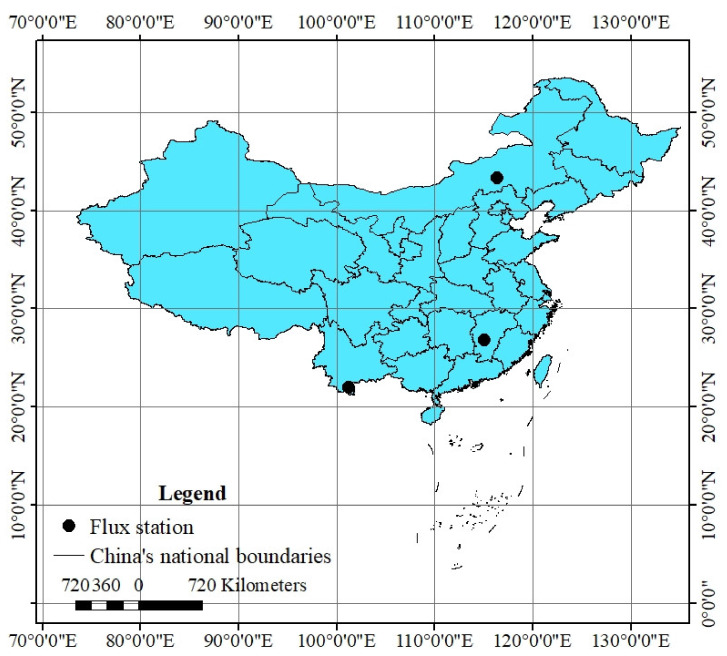
Location of monitoring stations for CO_2_ flux in temperate grassland, subtropical artificial coniferous forest, and tropical rain forest ecosystems.

The Xilinhot (XLHT) flux station (116°24′14.4″ E, 43°19′31.8″ N) is located in a temperate grassland (TG) ecosystem. The soil type is dark chestnut soil, and the texture is light loam. The dominant plants include *Leymus chinensis*, *Agropyron cristatum*, *Stipa grandis*, *Cleistogenes squarrosa*, and *Carex duriuscula*. Canopy height is about 0.5 m, and leaf area index is about 1.4 m^2^ m^−2^. The Qianyanzhou (QYZ) flux station (115°4′ E, 26°44′ N) is located in a subtropical artificial coniferous forest (SACF) ecosystem, with an average altitude of 110.8 m. The soil type is red soil. Existing forests are mainly plantations that were planted in 1985. The main plants are *Pinus massoniana*, *Pinus elliottii*, and *Cunninghamia lanceolata*. The average canopy height is 12 m, and leaf area index is 3.5 m^2^ m^−2^. The Xishuangbanna (XSBN) flux station (101°12′44″ E, 21°57′32″ N) is located in a tropical rain forest (TRF) ecosystem. The soil type is brick red soil, pH < 5, and the dominant plants are *Pometia tomentosa* and *Terminalia myriocarpa*. The average canopy height is 18.6 m [32,33,34] (Figure 1).

### 2.2. Data Collection

Initially, the CO_2_ flux and meteorological data of various ecosystems were collected. Then, the data of these ecosystems were classified according to the instruments used for monitoring to reduce errors caused by different instrument models. Finally, the temperate grassland ecosystem in Xilinhot, subtropical artificial coniferous forest ecosystem in Qianyanzhou, and tropical rain forest ecosystem in Xishuangbanna were selected based on the frequency and duration of monitoring. The three ecosystems were universal and accurate in each climate zone. The RE, GEE, NEE, soil and air temperature, soil and air moisture, and photosynthetically active radiation data of Xilinhot (2004–2010), Qianyanzhou (2003–2010), and Xishuangbanna (2003–2010) were derived from “A dataset of carbon and water fluxes over Xilinhot temperate steppe in Inner Mongolia (2003–2010)”, “An observation dataset of carbon and water fluxes of artificial coniferous forests in Qianyanzhou (2003–2010)”, and “A dataset of carbon, water and energy fluxes observed in Xishuangbanna tropical seasonal rain forest from 2003 to 2010” [32,33,34]. The information of the instruments used by flux stations is shown in Table 1. The layout of the instruments is described in Table 2.

### 2.3. Temperature Index

In this study, the temperature indicators were monthly maximum temperature (*Tmax*), monthly minimum temperature (*Tmin*), monthly temperature range (*Tef*), monthly average temperature (*Tave*), and monthly accumulated temperature (*AcT*). *Tmax* is the monthly maximum temperature; *Tmin* is the monthly minimum temperature; *Tef* is the difference between *Tmax* and *Tmin*; *Tave* is the monthly average temperature; *AcT* is the sum of the daily average temperature greater than 10 °C.

## 3. Results

### 3.1. Interannual Variation of Ecosystem Respiration

**Figure 2:**
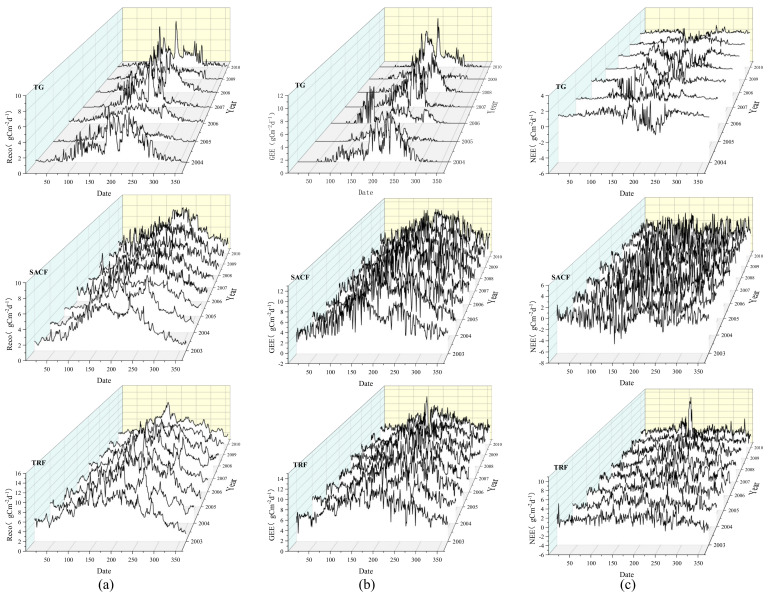
Inter−annual variation of ecosystem respiration (**a**), gross ecosystem exchange (**b**), and net ecosystem exchange (**c**) in temperate grassland (2004–2010), subtropical artificial coniferous forest (2003–2010), and tropical rain forest (2003–2010). Note: TG is temperate grassland, SACF is subtropical artificial coniferous forest, TRF is tropical rain forest.

As shown in Figure 2a, in TG, the maximum value of RE usually occurred from June to August. The average maximum value from 2003 to 2010 was 5.42 gC m^−2^ d^−1^. The minimum value (close to 0) generally occurred between December and February. The range of the difference between the maximum and the minimum was about 3.10–8.36 gC m^−2^ d^−1^. In SACF, the maximum value of RE usually occurred from May to August, the average maximum RE was 39.29% lager than TG. The minimum value generally occurred in December and January, with an average of 0.5 gC m^−2^ d^−1^. The range of the difference between the maximum and the minimum was about 4.95 to 7.56 gC m^−2^ d^−1^. In TRF, the range of the difference between the maximum and the minimum was about 6.68 to 12.18 gC m^−2^ d^−1^. The average maximum RE value of TRF was 63.84% and 128.23% larger than SACF and TG, respectively, and the average minimum value was also considerably larger than that of SACF and TG. In the three regions, the maximum and minimum value of RE appeared in similar periods, but the range of the difference between the maximum and the minimum was largest in TRF, at 110.73% and 4.56% larger than SACF and TG, respectively.

### 3.2. Interannual Variation of Gross Ecosystem Exchange

As shown in Figure 2b, in TG, the maximum value of GEE usually occurred between June and August, with an average of 6.57 gC m^−2^ d^−1^. The minimum value (close to 0) generally occurred between November and February. The range of the difference between the maximum and the minimum was 2.65 to 10.66 gC m^−2^ d^−1^. In SACF, the maximum GEE value usually occurred between May and August, and the average maximum was 66.97% larger than TG. The minimum GEE value generally occurred in December and January, with an average of 0.5 gC m^−2^ d^−1^. The range of the difference between the maximum and the minimum was about 8.83 to 11.63 gC m^−2^ d^−1^. In TRF, the range of the difference between the maximum and the minimum was about 7.49 to 11.23 gC m^−2^ d^−1^. The average maximum TRF value was 11.49% and 86.15% larger than that of SACF and TG, respectively, and the average minimum value was also considerably larger than that of SACF and TG. The maximums and minimums of the three regions were similar, but the range of the difference between the maximum and the minimum was largest in TG, at 186.07% and 114.17% larger than SACF and TRF, respectively. The interannual variation of GEE was similar to that of RE, increasing initially and then decreasing in December and January.

### 3.3. Interannual Variation of Net Ecosystem Exchange

As shown in Figure 2c, in TG, the ecosystem was a carbon source from January to December in 2005 and 2009, and there were 336 and 361 days as a carbon source in 2005 and 2009, respectively. From 2003 to 2010, except May–July 2003, June–September 2004, March–April 2006, May–August 2007, June–September 2008, and May–August 2010, the TG was a carbon source in all other months. In 2004, 2006, 2007, 2008, and 2010, the TG acted as a carbon source for 296, 298, 298, 290, and 311 days, respectively. Therefore, TG was a carbon source overall; RE was always greater than GEE, and there was no clear trend to identify in the inter-annual NEE in the study period. The average NEE of TG from 2004 to 2010 was 91.42 gC m^−2^ d^−1^, and the TG acted as a carbon source for an average of 313 days per year.

The SACF ecosystem acted as a carbon sink every month from 2003 to 2010, and there were only 67, 72, 88, 80, 80, 77, 97, and 112 days as carbon source in each year, respectively. The average NEE of SACF from 2003 to 2010 was −455.57 gC m^−2^ d^−1^, and it acted as a carbon source for an average of 84 days per year. From the beginning to the end of each year, the NEE first decreased and then increased.

In TRF, from 2003 to 2010, except March–August 2003, March–June 2004, May–August 2005, July and October 2006, June–July 2007, March–August 2008, March–August 2009, and April–June 2010, all the other months were carbon sinks. The SACF ecosystem acted as a carbon source for 151, 121, 117, 119, 104, 150, 127, and 79 days in each year, respectively. Therefore, the TRF was a carbon sink overall; GEE was always greater than RE and there was no obvious interannual change in NEE. The average NEE of TRF from 2003 to 2010 was −127.31 gC m^−2^ d^−1^, and TR acted as a carbon source for an average of 121 days per year.

The lowest annual average was SACF, which was 245.37% and 257.84% smaller than TG and TRF, respectively. SACF also had the least number of days as carbon source each year, at 73.16% and 30.58% less than for TG and TRF, respectively. The NEE represents the addition of RE and GEE; however, it showed no major trends compared with RE and GEE data alone.

### 3.4. Redundancy Analysis of CO_2_ Flux and Meteorology

#### 3.4.1. Redundancy Analysis of CO_2_ Flux and Temperature

**Figure 3:**
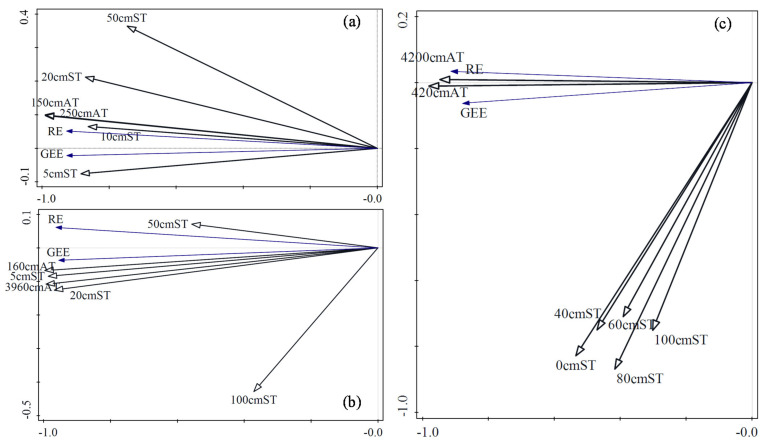
Redundancy analysis on temperature with ecosystem respiration and gross ecosystem exchange in temperate grassland (**a**), subtropical artificial coniferous forest (**b**), and tropical rain forest (**c**). Note: RE is ecosystem respiration, GEE is gross ecosystem exchange, AT is air temperature, ST is soil temperature, the number in front of ST and AT is the distance from the ground.

Plant photosynthesis and soil respiration are affected by temperature, and increases in temperature can correspondingly increase the rate of photosynthesis and respiration. However, soil temperature and air temperature have different effects on RE and GEE, and this can vary among locations. In this study, redundancy analysis (RDA) was used to calculate the effects of temperature at different locations on RE and GEE in different climatic zones and ecosystems (Figure 3). In TG, the air temperature at two locations and the soil temperature at four depths were analyzed. The air temperature 150 cm above the ground had the greatest impact on RE and GEE, and RE and GEE increased with the increase in temperature. In SACF, the air temperature was analyzed at two locations, and the soil temperature was measured at four depths. The air temperature near the ground had the greatest impact on RE and GEE, and RE and GEE increased with the increase of temperature. In TRF, the air temperature was analyzed at two locations, and the soil temperature at five depths, and the results were the same as those of SACF.

Figure 3 also shows that in various depths of soil, the soil temperature closer to the surface had the greatest impact on RE and GEE. The air temperature closer to the surface also had the greatest impact on RE and GEE. However, the influence of air temperature on RE and GEE was greater than that of soil temperature.

#### 3.4.2. Redundancy Analysis of CO_2_ Flux and Moisture

**Figure 4:**
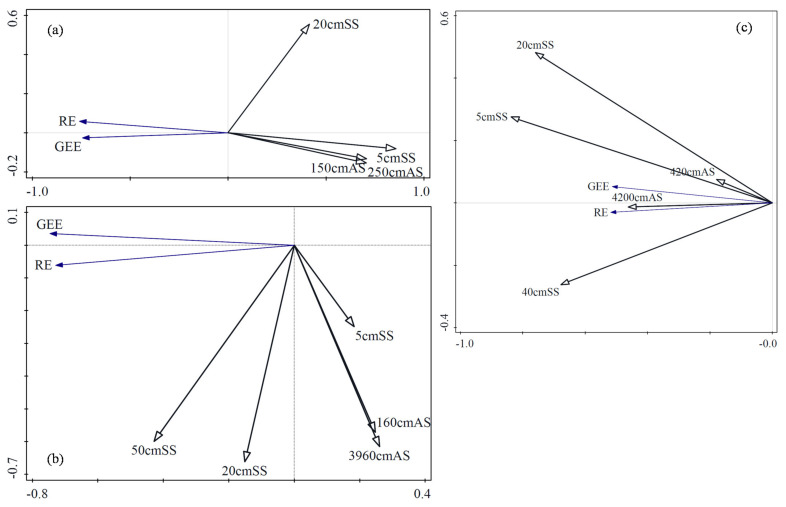
Redundancy analysis of moisture with ecosystem respiration and gross ecosystem exchange in temperate grassland (**a**), subtropical artificial coniferous forest (**b**), and tropical rain forest (**c**). Note: RE is ecosystem respiration, GEE is gross ecosystem exchange, AS is air moisture, SS is soil moisture, the number in front of SS and AS is the distance from the ground.

Moisture is also one of the major factors affecting photosynthesis and respiration. An optimal moisture content is conducive to plant growth and carbon fixation. More photosynthesis products are transferred to the soil, which also increases the intensity of soil respiration. However, too much water also inhibits the growth of plants and reduces the rate of soil aerobic respiration, leading to an increase in anaerobic respiration. In this study, the influence of moisture at different locations on RE and GEE was calculated through RDA (Figure 4). In TG, air moisture was analyzed at two locations and soil moisture at two depths. The soil moisture at 5 cm had the greatest impact on RE and GEE, and RE and GEE decreased with the increase in moisture. In SACF, air moisture was analyzed at two locations and soil moisture at three depths. The soil moisture at 50 cm had the greatest impact on RE and GEE. RE and GEE decreased with the increase in air moisture and soil moisture at 5 cm, and increased with the increase in soil moisture at 20 and 50 cm. In TRF, air moisture was analyzed at two locations and soil moisture at three depths. The soil moisture at 5 cm had the greatest impact on RE and GEE, and RE and GEE increased with the increase in moisture.

The impact of moisture on RE and GEE varied among different climatic zones and ecosystems. In TG, increased moisture reduced RE and GEE. In SACF, increased air moisture and soil moisture at 5 cm reduced RE and GEE, but the increase in soil moisture at 20 and 50 cm increased RE and GEE. In TRF, the increase in moisture increased the RE and GEE.

#### 3.4.3. The Main Factors Influencing Gross Ecosystem Exchange and Ecosystem Respiration

**Figure 5:**
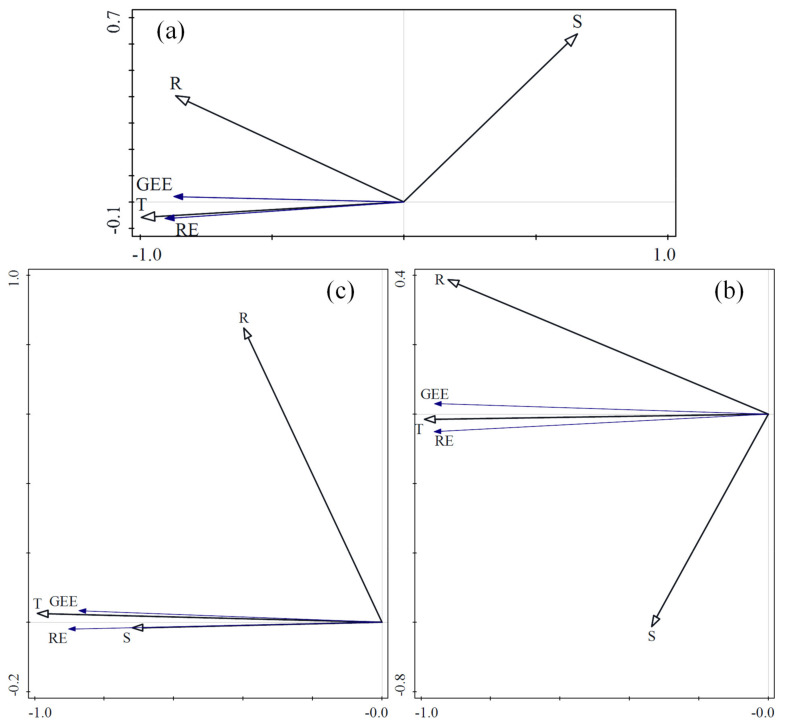
Redundancy analysis of temperature, moisture, photosynthetically active radiation, ecosystem respiration, and gross ecosystem exchange in temperate grassland (**a**), subtropical artificial coniferous forest (**b**), and tropical rain forest (**c**). Note: RE is ecosystem respiration, GEE is gross ecosystem exchange, T is temperature, S is moisture, R is photosynthetically active radiation.

In addition to temperature and moisture, photosynthetically active radiation is also an important component of climate change. Therefore, based on the results of Section 3.4.1 and Section 3.4.2, we selected the temperature and moisture data that had the greatest impact on RE and GEE. Then, combined with photosynthetically active radiation data, the main influencing factors of RE and GEE were determined by RDA (Figure 5). In the three ecosystems, temperature was the main factor influencing RE and GEE, and RE and GEE increased with the increase in temperature and photosynthetically active radiation. In TG and SACF, the influence of photosynthetically active radiation on RE and GEE was greater than that of moisture, but in TRF, the influence of moisture on RE and GEE was greater than that of photosynthetically active radiation.

### 3.5. Mathematical Model Construction

**Figure 6:**
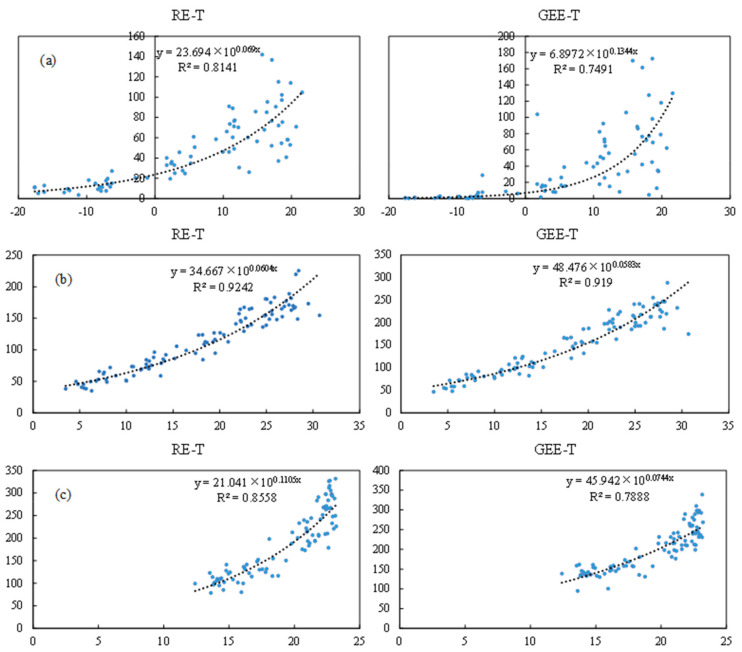
Mathematical modeling of temperature with ecosystem respiration and gross ecosystem exchange in temperate grassland (**a**), subtropical artificial coniferous forest (**b**), and tropical rain forest (**c**). Note: RE is ecosystem respiration, GEE is gross ecosystem exchange.

In the three ecosystems, temperature was the main factor influencing RE and GEE. Climate warming enhances ecosystem CO_2_ flux, which will inevitably lead to further climate change, thus forming a positive feedback loop. Therefore, mathematical models were constructed to assess the relationship between CO_2_ flux and temperature (Figure 6). In TG, the logarithmic model and power model were unsuitable for the test because the temperature data contained negative values. Therefore, in the linear model and the exponential model, the exponential model was subjected to the best fitting effect. In SACF and TRF, the temperature data contained all positive values, and thus the linear model, exponential model, logarithmic model, and power model fitting were included, but the fitting effect of the exponential model remained the best. This result is inconsistent with that of some previous studies but supported some other studies. This could be because the scale of the data was different [35,36]. The data selected in this study were on a monthly scale to highlight the cumulative effect of temperature on RE and GEE.

### 3.6. Relationship between CO_2_ Flux and Temperature Index

**Figure 7:**
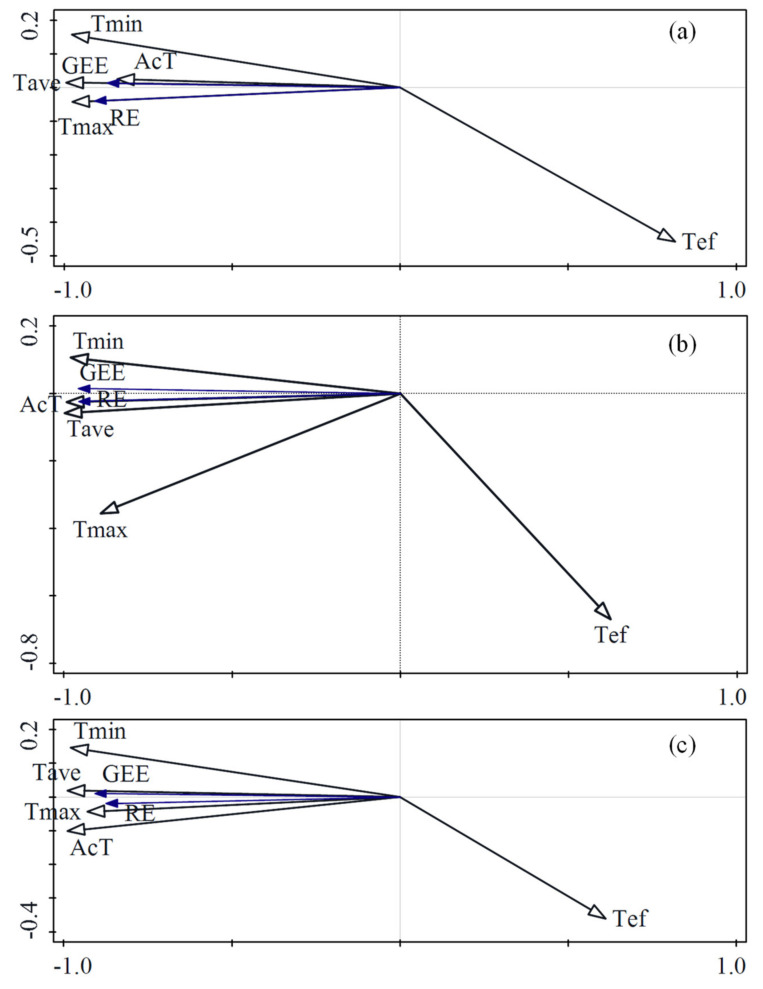
Redundancy analysis of temperature index with ecosystem respiration and gross ecosystem exchange in temperate grassland (**a**), subtropical artificial coniferous forest (**b**), and tropical rain forest (**c**). Note: RE is ecosystem respiration, GEE is gross ecosystem exchange.

**Figure 8:**
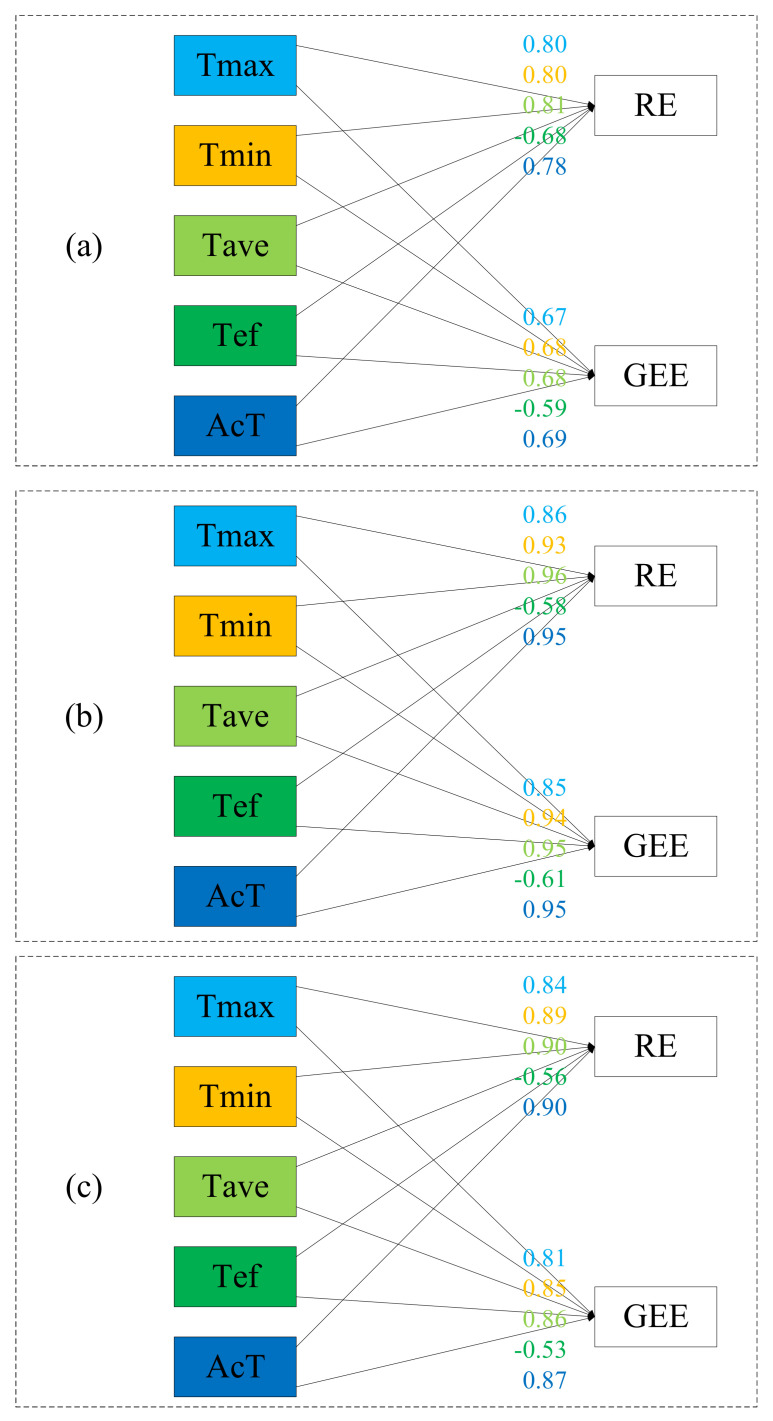
Correlation analysis of temperature index with ecosystem respiration and gross ecosystem exchange in temperate grassland (**a**), subtropical artificial coniferous forest (**b**), and tropical rain forest (**c**). Note: RE is ecosystem respiration, GEE is gross ecosystem exchange; the number is correlation coefficient; the above correlation coefficients are all found to be statistically significant at the *p* = 0.01 level; the same color corresponds to the corresponding coefficient.

The above results have shown the great influence of temperature on CO_2_ flux; however, for further detail, this next analysis considers the following different characteristics of temperature: *Tmax*, *Tmin*, *Tave*, *Tef*, and *AcT* (Figure 7 and Figure 8). Figure 7 shows that *Tave* was the main influencing factor of RE and GEE in TG. With the increase in *Tave*, *Tmax*, *Tmin*, and *AcT*, GEE and RE increased, and there was a negative correlation between *Tef* and RE or GEE. In SACF, *Tave* was also the main factor influencing RE and GEE, and RE and GEE were positively correlated with *Tave*, *Tmin*, *Tmax*, and *AcT* but negatively correlated with *Tef*. In TRF, *AcT* was the main influencing factor of RE and GEE. Similarly, *Tave*, *Tmax*, *Tmin*, and *AcT* were positively correlated with RE and GEE, and *Tef* was negatively correlated with RE and GEE.

Figure 8 presents the correlation analysis of temperature index and RE and GEE, and the above correlation coefficients were all found to be statistically significant at the *p* < 0.05 level. In the three ecosystems, the correlation coefficients of all temperature indices with RE and GEE were consistent with the RDA results, and the correlation coefficient of *Tave* with RE and GEE was the largest in TG and SACF, and the correlation coefficient of *AcT* with RE and GEE was the largest in TRF. These results provide a good verification of RDA.

In summary, in the three ecosystems, the positive and negative effects of temperature indicators on RE and GEE were the same, but the main influencing factor of TRF was *AcT*, while the main influencing factor of TG and SACF was *Tave*. These findings indicate that accumulated temperature has the strongest effect on CO_2_ flux.

## 4. Discussion

### 4.1. Evolution Characteristics, Similarities, and Differences of CO_2_ Fluxes in Different Ecosystems

Temperature, precipitation, radiation, and many other factors vary among climate zones, leading to differences in soil temperature, moisture, texture, pores, nutrients, and other factors, thus forming different ecosystems. Continued climate change will change the characteristics of temperature, precipitation, and radiation, and affect the ecosystem carbon cycle. Changes in the carbon cycle will continue to affect climate change, thus forming a positive feedback loop.

In the three ecosystems, the maximum values of RE and GEE were observed in June and July. During this period, the temperature is high, vegetation grows rapidly, photosynthetic enzyme activity is stimulated [37], thereby enhancing the photosynthetic process [38,39,40], the amount of CO_2_ sequestration increased considerably, but soil respiration also increased, leading to a significant increase in RE. Because of the differences in climate and plant types, the average maximum value of RE and GEE in TRF were larger than in SACF and TG. Furthermore, the minimum values of RE and GEE occurred at around December and January in the three ecosystems. During this period, vegetation is mainly in a dormant stage and soil respiration has attenuated, resulting in a significant decrease in RE and GEE [41]. Similarly, because of the differences in climate and vegetation types, the average minimum values of RE and GEE in TRF were significantly larger than those of SACF and TG. The range of the difference between the maximum RE and the minimum RE was larger than that of SACF and TG, but the range of GEE in TG was larger than that of SACF and TRF. This shows that the high temperature of TRF all year round enhances its CO_2_ sequestration capacity and respiration intensity, and since precipitation is relatively sufficient, the probability of soil respiration being inhibited is high. However, in TG, there was lower vegetation diversity and a lower biomass. Compared with TRF and SACF, the CO_2_ sequestration capacity in TG was more unstable. Therefore, under the influence of climate change, the ability of TG to resist environmental change can be considered weak.

In the three ecosystems, the inter-annual changes of RE and GEE have significant regularities, but NEE has no significant regularities. This may be because the changes in temperature, moisture, and photosynthetically active radiation have different effects on soil respiration and plant photosynthesis, and the amplitude and frequency of changes in environmental factors likewise have different effects. At present, TG is functioning as a carbon source overall, whereas SACF and TRF function as carbon sinks.

### 4.2. Similarities and Differences of Influencing Factors in Different Ecosystems

Temperature is an important factor affecting soil respiration and plant photosynthesis, but temperature at different locations showed different effects on respiration and photosynthesis. Figure 3 shows that the influence of air temperature on photosynthesis was greater than that of soil temperature. This indicates that the photosynthesis of plants is more directly affected by air temperature. Furthermore, GEE increased with the increase in temperature, which shows that the air temperature did not exceed the thermal limit temperature of photosynthesis, and the increase in temperature still contributed to the enzymatic reaction of photosynthesis. However, soil respiration is typically considered more directly affected by soil temperature, but this study shows that air temperature had a greater impact on RE than soil temperature. This indicates that the autotrophic respiration of roots and rhizosphere microorganisms in the study area is the main component of soil respiration, and soil respiration is mainly affected by the amount of photosynthesis products transported into the soil.

In the three ecosystems, temperature had the same impact on RE and GEE, the air temperature closer to surface had a greater impact on RE and GEE, and the impact of moisture on RE and GEE was very different. In TG and TRF, soil moisture at 5 cm had the greatest impact on RE and GEE. In TG, RE and GEE decreased with the increase in soil moisture at 5 cm, but in TRF, RE and GEE increased with the increase in soil moisture at 5 cm. In SACF, soil moisture at 50 cm had the greatest impact on RE and GEE. RE and GEE increased with the increase in soil moisture at 20 and 50 cm, and decreased with the increase in soil moisture at 5 cm. The plant roots in the TG ecosystem are mainly distributed in the upper soil, and evaporation is low, leading to sufficient moisture in the upper soil. With the increase in soil moisture, the oxygen gradually decreases, and autotrophic respiration is inhibited [42], which is not conducive to vegetation growth. Therefore, the increase in the frequency and intensity of heavy rains caused by climate change will increase the adverse impact on TG. However, in such a water-rich ecosystem, improvements in the leaf area index, photosynthetic enzyme activity, rhizobia formation, soil phosphorus and nitrogen content, and water–heat relationship [43] are more likely to stimulate microbial activities [44,45], so as to provide sufficient nutrients for vegetation growth [46] and enhance the vegetation absorption of CO_2_. Additionally, TRF is associated with high temperatures and high evaporation. The increase in water may change soil water holding capacity and availability [46,47], affect the structure and activity of the microbial community, improve soil fertility [37,48], be conducive to the growth of plants, and improve plants’ CO_2_ sequestration abilities. The amount of photosynthesis products transported into the soil also increases, which further improves the intensity of soil respiration. In addition, in SACF, soil moisture at 50 cm had the greatest impact on RE and GEE, indicating that plant roots are mainly distributed in the top 50 cm of soil, and the moisture at 50 cm is lower than the requirement for plant growth. This could be because the soil at 50 cm has a poor ability to hold water. With the increase in soil moisture at 5 cm from the surface, oxygen entering the soil is hindered, which is not conducive to autotrophic respiration. Moreover, the increase in secretions produced by root anaerobic respiration is not conducive to plant growth, resulting in reduced CO_2_ sequestration capacity.

After comparing the effects of temperature, moisture, and photosynthetically active radiation on RE and GEE, we found that temperature was the main influencing factor, which is consistent with many studies [49,50]. However, in some plant photosynthesis and respiration models [51,52,53,54], GEE is mainly calculated based on light and RE is mainly calculated based on temperature, which is different from the results of this paper. This shows that when calculating RE and GEE in models, indicators at different locations may require different weights. As the climate changes, temperature indicators should be paid more attention. Although light and radiation are the main factors affecting plant photosynthesis, the conditions of precipitation and light in the study areas can support the normal growth of vegetation, therefore, an appropriate increase in temperature will further enhance the intensity of photosynthesis and respiration. The exponential model exhibited the best fit between temperature and RE and GEE, indicating that as the temperature continues to increase, RE and GEE may surge, but if the soil matrix of different ecosystems is different, the response of CO_2_ flux to climate change may have different stages [55]. Therefore, the speed of reaction after the surge requires further research.

### 4.3. Relationship between CO_2_ Flux and Climate Change in Different Ecosystems

Climate change is associated not only with an increase in temperature but also with extreme weather such as heavy rains, floods, waterlogging, droughts, high temperatures, and sudden changes in droughts and floods. An analysis of the relationship between various temperature indicators and CO_2_ flux revealed that with the increase in *Tave*, *Tmax*, *Tmin*, and *AcT*, RE and GEE increased in the three climate zones. This indicates that the increase in temperature may increase the decomposition of SOM, change the activity of extracellular enzymes, thereby changing the strategy of the microbial community to obtain nutrients, control the biogeochemical cycle of soil nutrients, and lead to an increase in CO_2_ emissions [49,50]; at the same time, the nutrients released by SOM decomposition and microbial activities are beneficial to plant growth, leading to enhanced photosynthesis and respiration. However, *Tef* is negatively correlated with RE and GEE, which indicates that the increase in extreme weather will increase the mortality of vegetation [56,57,58] and reduce photosynthesis and respiration [59]. In TG and SACF, *Tave* was the main influencing factor for RE and GEE. In TRF, *AcT* was the main influencing factor for RE and GEE. This indicates that when the temperature can support plant development, plants can grow well, which is not a short process. Although all kinds of extreme weather can arrest or slow the growth process of plants and have an impact on CO_2_ emissions, the ecosystem itself has a certain degree of resilience and can recover from the impact of short-term extreme weather. Therefore, changes in RE and GEE mainly depend on the plant’s developmental state, extreme weather caused by climate change may not be the main impact on ecosystem CO_2_ emissions, and a slow increase in temperature may have a greater impact.

Therefore, moderate increases in temperature are conducive to the growth and development of plants and promotes photosynthesis and respiration. Meanwhile, the increase in vegetation biomass will increase litter and root exudates in the soil and may change the composition of the microbial community [60,61]. However, extreme weather such as torrential rain, flood, waterlogging, and drought inhibits the growth process of plants and will inevitably have an adverse effect on photosynthesis and respiration. The increase in precipitation may have a beneficial effect on TRF, but it may have a negative effect on TG and SACF. Moreover, when plant growth and development are restricted, the absorption of soil nutrients by roots will decrease, and respiration will consume more soil nutrients, leading to an increase in CO_2_ emissions, further aggravating climate change and forming a vicious circle. Correspondingly, climate change can be expected to negatively impact TG and SACF, whereas TRF is more likely to be resistant to climate change effects.

## 5. Conclusions

The interactions between CO_2_ flux and climate change vary significantly among different ecosystems. These variations lead to different resistance responses to climate change. In this study, the inter-annual variation characteristics of RE, GEE, and NEE were analyzed for the temperate grassland ecosystem in Xilinhot (2004–2010), the subtropical artificial coniferous forest ecosystem in Qianyanzhou (2003–2010), and the tropical rain forest ecosystem in Xishuangbanna (2003–2010). The main factors of climate change affecting ecosystem CO_2_ flux were identified by redundancy analysis, and the impact mechanism of climate change on ecosystem CO_2_ flux was discussed. This research shows that from 2003 to 2010, TG was a carbon source in Xilinhot, whereas SACF and TRF were carbon sinks in Qianyanzhou and Xishuangbanna, respectively. The average maximum value of RE is 5.42 gC m^−2^ d^−1^ in TG, which is 39.29% and 128.23% less than SACF and TRF, respectively. The average maximum value of GEE is 6.57 gC m^−2^ d^−1^ in TG, which is 66.97% and 86.15% less than SACF and TRF, respectively. The influence of air temperature on RE and GEE in the three ecosystems was greater than that of soil temperature, and the air temperature closer to surface had a greater impact on RE and GEE. Soil respiration was mainly affected by the amount of photosynthesis products transported into the soil. The influence of soil moisture on RE and GEE in the three ecosystems was greater than that of air moisture. The evaporation of TRF was large, and the increase in soil moisture at 5 cm was beneficial to plant growth, leading to a significant increase in RE and GEE. However, the evaporation of TG and SACF was relatively small, and the increase in soil moisture at 5 cm may reduce available oxygen and inhibit plant growth, resulting in a decrease in RE and GEE.

In the three ecosystems, temperature was the main influencing factor on RE and GEE. RE and GEE were positively correlated with *Tave*, *AcT*, *Tmax*, and *Tmin* and negatively correlated with *Tef*. The influences of *Tave* and *AcT* on RE and GEE were larger than those of *Tmax* and *Tmin*. The extreme weather brought about by climate change may arrest or inhibit plant growth, increase CO_2_ emissions, and further aggravate the process of climate change. A brief increase in temperature may be beneficial to plant growth and promote photosynthesis and respiration; however, a continuous increase in temperature may cause a surge in RE and GEE. The consequences of this surge and the behavior of the RE and GEE after the surge require further research.

Therefore, the basis of China’s ecological conservation in the future is preserving temperate grassland ecosystems. Moreover, increasing vegetation coverage is an effective way to enhance the stability of near-surface air temperature and improve the resilience of ecosystems’ responses to climate change.

## Figures and Tables

**Table 1:** Instrument information for measuring meteorological and CO_2_ fluxes.

Parameters	Instrument Model	Production Company	Country
Ecosystem respiration	LI-7500	Campbell Scientific Inc	USA
Gross ecosystem exchange	LI-7500	Campbell Scientific Inc	USA
Photosynthetically active radiation	LI190SB	LI-COR	USA
Air temperature	HMP45C	Vaisala	USA
Air moisture	HMP45C	Vaisala	USA
Soil temperature	DR	Campbell Scientific	USA
Soil moisture	DR	Campbell Scientific	USA

The measuring instruments at the three stations are the same.

**Table 2:** Instrument layout and monitoring frequency.

Parameters	Frequency (min)	Position (m Above Ground Level)		
		XLHT	QYZ	XSBN
Ecosystem Respiration	30	2.5	39.6	42
Gross Ecosystem Exchange	30	2.5	39.6	42
Photosynthetically active radiation	30	1.5	39.6	7
Air temperature	30	1.5 and 2.5	1.6 and 39.6	4.2 and 42
Air moisture	30	1.5 and 2.5	1.6 and 39.6	4.2 and 42
Soil temperature	30	0.05, 0.1, 0.2, 0.5, and 1.0 (m below ground)	0.05, 0.1, 0.2, 0.5, and 1.0 (m below ground)	0.05, 0.1, 0.2, 0.5, and 1.0 (m below ground)
Soil moisture	30	0.05, 0.2, and 0.5 (m below ground)	0.05, 0.2, and 0.5 (m below ground)	00.05, 0.2, and 0.5 (m below ground)

XLHT is the Xilinhot, QYZ is the Qianyanzhou, XSBN is the Xishuangbanna.

## Data Availability

Not applicable.
